# Gray Matter Sampling Differences Between Subdural Electrodes and Stereoelectroencephalography Electrodes

**DOI:** 10.3389/fneur.2021.669406

**Published:** 2021-04-27

**Authors:** Mohamed Tantawi, Jingya Miao, Caio Matias, Christopher T. Skidmore, Michael R. Sperling, Ashwini D. Sharan, Chengyuan Wu

**Affiliations:** ^1^Department of Radiology, Jefferson Integrated Magnetic Resonance Imaging Center, Thomas Jefferson University, Philadelphia, PA, United States; ^2^Department of Neurosurgery, Thomas Jefferson University, Philadelphia, PA, United States; ^3^Department of Neurology, Thomas Jefferson University, Philadelphia, PA, United States

**Keywords:** intracranial monitoring, epileptogenic zone, stereoelectroencephalography, depth electrodes, subdural grid, intracranial electrodes

## Abstract

**Objective:** Stereoelectroencephalography (SEEG) has seen a recent increase in popularity in North America; however, concerns regarding the spatial sampling capabilities of SEEG remain. We aimed to quantify and compare the spatial sampling of subdural electrode (SDE) and SEEG implants.

**Methods:** Patients with drug-resistant epilepsy who underwent invasive monitoring were included in this retrospective case-control study. Ten SEEG cases were compared with ten matched SDE cases based on clinical presentation and pre-implantation hypothesis. To quantify gray matter sampling, MR and CT images were coregistered and a 2.5mm radius sphere was superimposed over the center of each electrode contact. The estimated recording volume of gray matter was defined as the cortical voxels within these spherical models. Paired *t*-tests were performed to compare volumes and locations of SDE and SEEG recording. A Ripley's K-function analysis was performed to quantify differences in spatial distributions.

**Results:** The average recording volume of gray matter by each individual contact was similar between the two modalities. SEEG implants sampled an average of 20% more total gray matter, consisted of an average of 17% more electrode contacts, and had 77% more of their contacts covering gray matter within sulci. Insular coverage was only achieved with SEEG. SEEG implants generally consist of discrete areas of dense local coverage scattered across the brain; while SDE implants cover relatively contiguous areas with lower density recording.

**Significance:** Average recording volumes per electrode contact are similar for SEEG and SDE, but SEEG may allow for greater overall volumes of recording as more electrodes can be routinely implanted. The primary difference lies in the location and distribution of gray matter than can be sampled. The selection between SEEG and SDE implantation depends on sampling needs of the invasive implant.

## Introduction

Approximately 1 third of patients with epilepsy become drug-resistant and may benefit from surgery (1–3). The main goal of the surgery is to resect the epileptogenic zone (EZ), while preserving the functional cortical areas. Localization of the EZ is initially assessed through a multimodal approach consisting of a clinical history, neuroimaging, neuropsychologic tests, and scalp video-EEG recordings. Unfortunately, such data is insufficient for localization in 30–50% of cases (4). In such cases, intracranial monitoring may be warranted for further delineation or clarification of the EZ (5).

Techniques for intracranial monitoring include implantation of subdural electrodes (SDE), which may be combined with depth electrodes; and stereoelectroencephalography (SEEG). SEEG is typically preferred for patients who have deep seated or MRI-occult lesions, have previously undergone surgery, or require bilateral exploration (6). Meanwhile, subdural grids are preferred for cortical lesions within the brain convexity, especially if close to an eloquent area as SDE would allow for easier cortical mapping (6, 7). SDE implantation was historically the most common method in the North America, but with the recent introduction of stereotactic robots, practice patterns have changed toward greater utilization of SEEG (8). In addition, the ability of SEEG to investigate the three-dimensional organization of ictal discharge has further motivated this transition. Studies have demonstrated a superior safety profile of SEEG, with an overall complication rate of 1.3% vs. the 3.5% seen with SDE (9–13). SEEG has also been associated with less perioperative pain and shorter recovery times (13, 14).

While procedural safety has been compared, there has been no direct metric for describing the efficacy of localization of the EZ – further complicated by the heterogeneity the patient population (15). While the efficacy of SEEG has been implicated in studies that report the number of patients who achieve seizure freedom after subsequent surgical intervention (16), the comparative efficacy of both techniques has been a matter of debate (9, 10, 17).

Ultimately, accurate localization of the EZ relies on several factors, including the preimplantation hypothesis and the interpretation of clinical data (18). Furthermore, the spatial sampling capabilities of each technique may also play a role in localization. An objective comparison of the spatial sampling between SDE and SEEG has yet to be performed. We therefore aimed to objectively assess the spatial sampling capabilities of both modalities by comparing estimated volumes of gray matter covered by SEEG and SDE implants.

## Materials and Methods

### Patient Inclusion and Data Acquisition

This study was approved by the local Institutional Review Board. Twenty consecutive patients with drug-resistant epilepsy who required long-term invasive monitoring (10 SEEG cases and 10 SDE cases without concurrent depth electrodes) were retrospectively included in this study. Each SEEG implant patient was matched with a historical control – a patient with a similar seizure semiology, seizure onset, and seizure spread hypothesis (based on Phase I evaluation) who underwent a SDE implantation. In all patients, the implants were placed on the side of the brain specified by the pre-implantation hypothesis, however, each pair of patients did not necessarily have their implants on the same side of the brain. Of note, our institution changed its practice of invasive monitoring almost entirely from SDE implants to SEEG implantations in 2015 (SDE is still used in <5% of cases where language mapping is deemed essential). As such, the SDE cases included in this study were managed before this switch.

As part of their routine clinical care, all subjects underwent preoperative T1-weighted magnetic resonance imaging (MRI), acquired from a 3.0T Philips Achieva MR scanner with an 8-channel head coil (FOV = 240 mm, voxel size 1.0 × 1.0 × 1.0 mm^3^, TR = 12ms, TE = 6ms), and post-implantation computed tomography (CT) acquired from Philips scanner (scan option HELIX, in-plane resolution 512 × 512, kVp = 120kV, mAs = 130–300mAs, slice thickness = 0.8–1.0mm).

### Electrode Localization

*FreeSurfer* was used to parcellate cortical and subcortical anatomy in each subject's native anatomical space based on T1-weighted MRI (19, 20). Each subject's post-implantation CT was registered to their preoperative MRI using *FSL* FLIRT with rigid body algorithms (21, 22). The coregistered preoperative MRI and post-implantation CT, as well as the brain mask image obtained from *FreeSurfer*, were loaded to the *iElectrodes* software package (23). This toolbox uses a graphical user interface for visual validation of contact localization and extracts the electrode contact coordinates (center of mass of each electrode contact) in a standard space. The coordinates were then transformed back to the subject's native space using the affine transformation matrix found in the image header of their preoperative MRI.

Since brain shift from SEEG implantation is insignificant (24), SEEG contacts were localized directly from the post-implantation CT coregistered to the preoperative MRI (23, 25). For each SEEG image set, minimal pneumocephalus (<1cc) was noted, which helped verify the absence of significant brain shift. Brain deformation from SDE implantation, however, is significant; and methods to correct for this phenomenon have been previously described to allow for accurate electrode localization (26, 27). Nevertheless, since the goal of this study was to quantify the interaction between gray matter and each electrode contact, the exact location of each electrode contact was of lesser importance. To correct for brain deformation and ensure SDE contacts were localized to the cortical surface, each SDE contact was projected along a radial path from the geometric center of either hemisphere to its smoothed pial surface obtained using the Local Gyrification Index technique in *FreeSurfer* (28).

### Recording Volume in Gray Matter

We aimed to quantify the gray matter sampled by the contacts of each modality. There is no consensus on the volume of spatial sampling of a single contact, or the proximate cortical area that contributes to a local field potential (LFP) – with estimates ranging from hundreds of micrometers to a few millimeters (29–31). Maling et al. simulated the spatial extent of LFPs recorded from deep brain stimulation electrodes and concluded that a sphere with a 2.4 mm radius best represents this spatial sampling (31). We therefore applied a model with a 2.5 mm radius to simulate the recording volume from each electrode contact. Inherently, this model was spherical for SEEG electrode contacts and a half sphere for SDE contacts (as these electrodes have a single two-dimensional recording surface). Summation of the volume of spatial sampling around each implanted contact then allowed for the calculation of a *total recording volume* for each subject.

**Figure 1:**
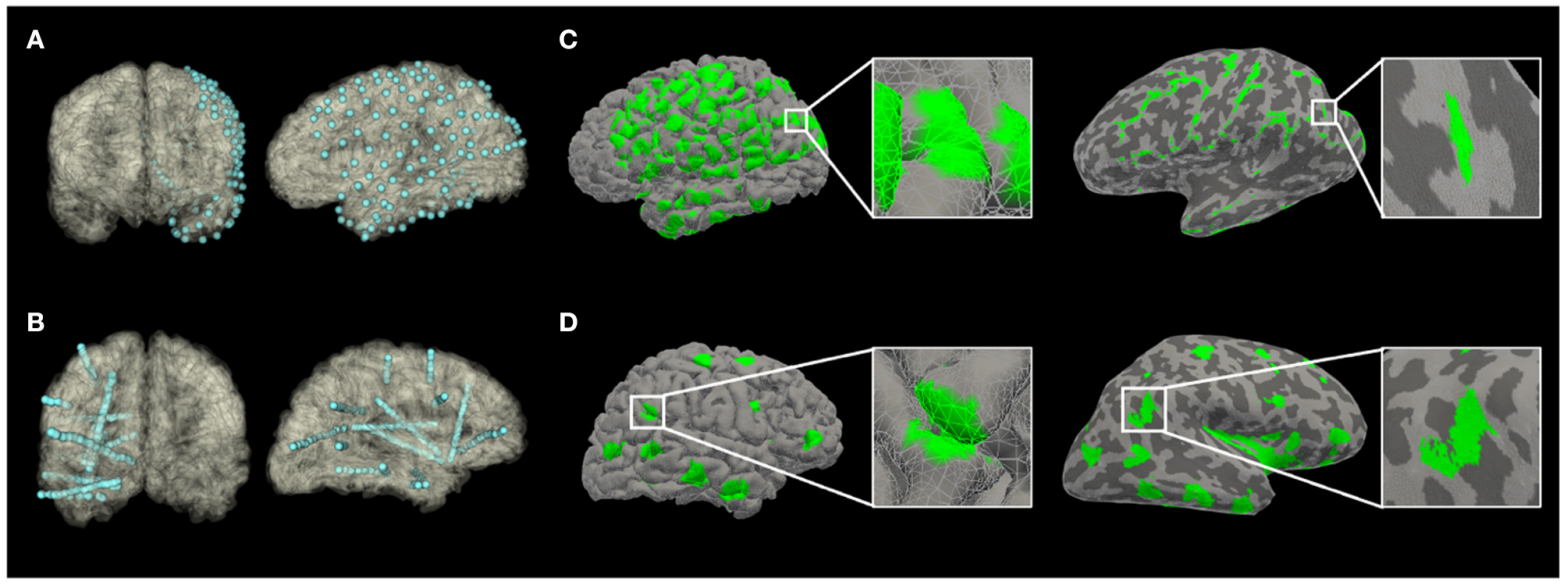
The recording volume of gray matter covered by implants, projected to an anatomical pial surface **(A,C)** or an inflated pial surface **(B,D)** of subjects in Pair 1 (**A,B** from Subject S1; **C,D** from Subject G1). On the inflated pial surface images, the lighter areas represent the gray matter over the convexity (crest of gyrus); while the darker areas represent the gray matter within sulci.

The gray matter of interest, consisting of the cerebral cortex, amygdala, and hippocampus, was obtained from *FreeSurfer* parcellations and grouped into 7 regions of interest (ROIs): cingulate, frontal, parietal, insular, lateral temporal, mesial temporal, and occipital cortex. To separate gyri from sulci, a smoothed white matter surface was obtained using the Local Gyrification Index technique in *FreeSurfer* (28). Gray matter outside the smoothed white matter surface was labeled as *gyri*; and gray matter within this surface was labeled as *sulci* (Figure 1). Using custom MATLAB scripts, a mask of each subject's recording volume was overlaid with their gray matter parcellation (with sulci/gyri labels) and ROIs. The *total gray matter volume recorded* was then calculated by summating the overlapping voxels between the recording volume mask and the gray matter parcellation. *Gray matter contacts* were defined as the number of contacts covering gray matter. From this value, the percentage of gray matter contacts and the average recording volume per gray matter contact was calculated for each subject. The percentage of sulcal coverage was also calculated for each subject.

Statistical analysis was performed using *GraphPad* Prism (v8.4.3). The D'Agostino & Pearson omnibus normality test was performed to test for normal distribution. A *t*-test was used to analyze data that passed the normality test, whereas a Wilcoxon test was used when variables failed the normality test. A paired *t*-test was performed for comparison of the number of gray matter contacts, the total volume of gray matter recorded, the average recording volume per gray matter contact, and the percentage of sulcal coverage between the pair-matched SEEG and SDE implants (32, 33). The Wilcoxon matched-pairs signed rank test was performed for the comparison of gray matter recording volume in each ROI between pair-matched SEEG and SDE implants. A two-tailed *p* < 0.05 was taken to be statistically significant.

### Quantification of Spatial Distribution of Contacts

We used the Ripley's K-function to map the distribution of the gray matter sampled by each modality. The Ripley's K-function can be used to describe spatial point patterns and can identify clustering, randomness, and regularity in distributions (34). We therefore used it to quantify the differences in electrode contact distribution between SEEG and SDE implants. The Ripley's K-function has been previously described in detail, but briefly, it represents a normalized average number of neighboring contacts as a function of distance from an arbitrary contact (35):

$$\begin{array}{l} K\left(d\right)\\ =\frac{Average\;number\;of\;contacts\;within\;a\;distance{\;}^{\prime }d^{\prime }\;of\;an\;arbitrary\;contact}{The\;total\;contact\;density} \end{array}$$

The numerator is calculated by averaging the number of neighboring contacts within a distance *d* (in millimeters) across all contacts of the implant. The denominator of total contact density serves a means of normalization and is calculated as follows:

$$\begin{array}{l} Total\;contact\;density\\ =\frac{Total\;number\;of\;contacts\;}{Minimum\;cubic\;volume\;containing\;all\;contacts} \end{array}$$

The variance of the estimated K-functions was calculated using bootstrap resampling over 100 simulations yielding 95% confidence intervals, which were then compared using a modification of the sum of squares' function (35). The null hypothesis was that the two sets of distribution are based on the same underlying distribution. For hypothesis testing, 5,000 random sets with the same number of distributions as the original sets were created using distributions from the original sets. A modified sum of squares' score was calculated for every random set, as well as the original sets. Consequently, the probability of the scores of the original sets being produced by the random sets under the null hypothesis was calculated with a *p* < 0.05. These calculations were performed in the MatLab-based RipleyGUI software package (35). A schematic visualizing the K-function calculation is shown in Supplementary Figure 1.

## Results

### Clinical Characteristics

Clinical details for all subjects in their matching pairs are outlined in Table 1.

**Table 1:** Subject Clinical Details. Each SEEG implant was matched with a SDE implant with a similar seizure semiology, seizure onset, and seizure spread hypothesis (based on Phase I evaluation).

		**SEEG**				**SDE**			
**Pair#**	**Pre-implantation hypothesis**	**Subject**	**Phase I results**	**Electrodes#**	**Contacts#**	**Subject**	**Phase I results**	**Electrodes Information**	**Contacts #**
1	Frontal, temporal, parietal, insula	S1	L temporal	15	180	G1	R temporoparietal	8 × 8 grid (1), 1 × 8 strips (8)	128
2	Bitemporal, L fronto-insular	S2	L anterior temporal	11	145	G2	L anterior temporal	1 × 8 strips (9)	72
3	Fronto-temporal	S3	L temporal	22	284	G3	L perirolandic	1 × 8 strips (22)	176
4	Bilateral fronto-temporal, R parietal	S4	R orbitofrontal	14	172	G4	R temporal	1 × 8 strips (17), 1 × 4 strip (1)	140
5	Left hemispheric	S5	L frontal	16	184	G5	L posterior temporal/occipital	8 × 8 grid (1), 1 × 8 strips (10), 1 × 6 strip (1)	150
6	Fronto-parietal, R insula	S6	R frontal	17	180	G6	R frontal	8 × 8 grid (1), 1 × 6 strips (3), 1 × 8 strips (6)	130
7	Frontal, temporal, parietal, occipital	S7	R fronto-temporal	20	298	G7	R fronto-temporal	1 × 8 strips (20), 1 × 4 strips (3)	172
8	Posterior temporal, frontal, occipital	S8	L anterior temporal	17	178	G8	L mid-temporal	4 × 6 grid (1), 1 × 8 strips (6)	72
9	Occipital, posterior temporal	S9	R temporoparietal	11	154	G9	L temporooccipital	4 × 5 grid (1), 1 × 4 strips (3), 1 × 8 strips (5)	72
10	Temporal	S10	L temporal	13	146	G10	L temporal	4 × 6 grid (1), 1 × 8 strips (7)	80

*The number of each grid/strip electrode implanted is shown in parentheses. M, male; F, female; L, left; R, right*.

### Electrode Coverage

There were 192 ± 54 (range: 145–298) SEEG electrode contacts and 119 ± 42 (range: 72–176) SDE contacts implanted per subject. SEEG implants had significantly lower percentage of gray matter contacts than SDE implants (*t* = 8.432, *p* < 0.0001); however, on average, there were more contacts covering gray matter and greater total volumes of gray matter recorded by SEEG than SDE (Table 2). Specifically, SEEG implants consisted of an average of 17% more gray matter contacts than SDE implants (*t* = 2.286, *p* = 0.0481); with eight of the ten SEEG subjects having more contacts covering gray matter than their SDE counterpart. With regards to the total volume of gray matter recorded, SEEG implants recorded on average of 20% more than SDE (*t* = 2.305, *p* = 0.0466); with eight of the ten subjects having a larger total volume of gray matter recorded with SEEG implants. The average percentage of sulcal coverage of SEEG implants was 78.2 ± 9.4%, which was significantly higher (*t* = 26.11, *p* < 0.0001) than that for SDE (0.93 ± 1.2%). There was no significant difference in recording volume by each gray matter contact between SEEG and SDE implants (*t* = 0.05727, *p* = 0.9556). A representation of the electrode contacts and an illustrative case of the recording volumes projected to the pial surface and an inflated pial surface is shown in Figure 1.

**Table 2:** Comparison of the number of gray matter contacts, the total volume of gray matter recorded, the average recording volume per gray matter contact, and the percentage of sulcal coverage between SEEG and SDE implants.

**Pair#**	**Number of gray matter contacts**			**Total volume of gray matter recorded by iEEG implant [mm**^****3****^**]**			**Recording volume per gray matter contact [mm**^****3****^**]**		**Sulci coverage percentage [%]**	
	**SEEG**	**SDE**	**Difference**	**SEEG**	**SDE**	**Difference**	**SEEG**	**SDE**	**SEEG**	**SDE**
1	137	121	16	3,518	3,384	134	25.7	28.0	81.0	1.21
2	118	71	47	3,899	2,181	1,718	33.0	30.7	89.9	0.18
3	203	165	38	5,646	4,601	1,045	27.8	27.9	74.2	1.59
4	98	135	−37	2,581	3,509	−928	26.3	26.0	82.1	0.26
5	141	142	−1	4,427	3,419	1,008	31.4	24.1	69.6	1.26
6	137	119	18	3,099	2,252	847	22.6	18.9	62.7	0.09
7	186	158	28	4,941	4,193	748	26.6	26.5	88.3	0.24
8	127	69	58	3,580	2,059	1,521	28.2	29.8	66.4	0.15
9	87	69	18	1,861	1,859	2	21.4	26.9	83.2	3.98
10	88	80	8	2,822	2,963	−141	32.1	37.0	84.5	0.30
Mean ± SD	132 ± 39	113 ± 38		3637 ± 1139	3042 ± 941		27.5 ± 3.9	27.6 ± 4.7	78.2 ± 9.4	0.93 ± 1.2
*P*-value	0.0481^*^			0.0466^*^			0.9556		*p* < 0.0001^*^	

* *Statistically significant result*.

**Figure 2:**
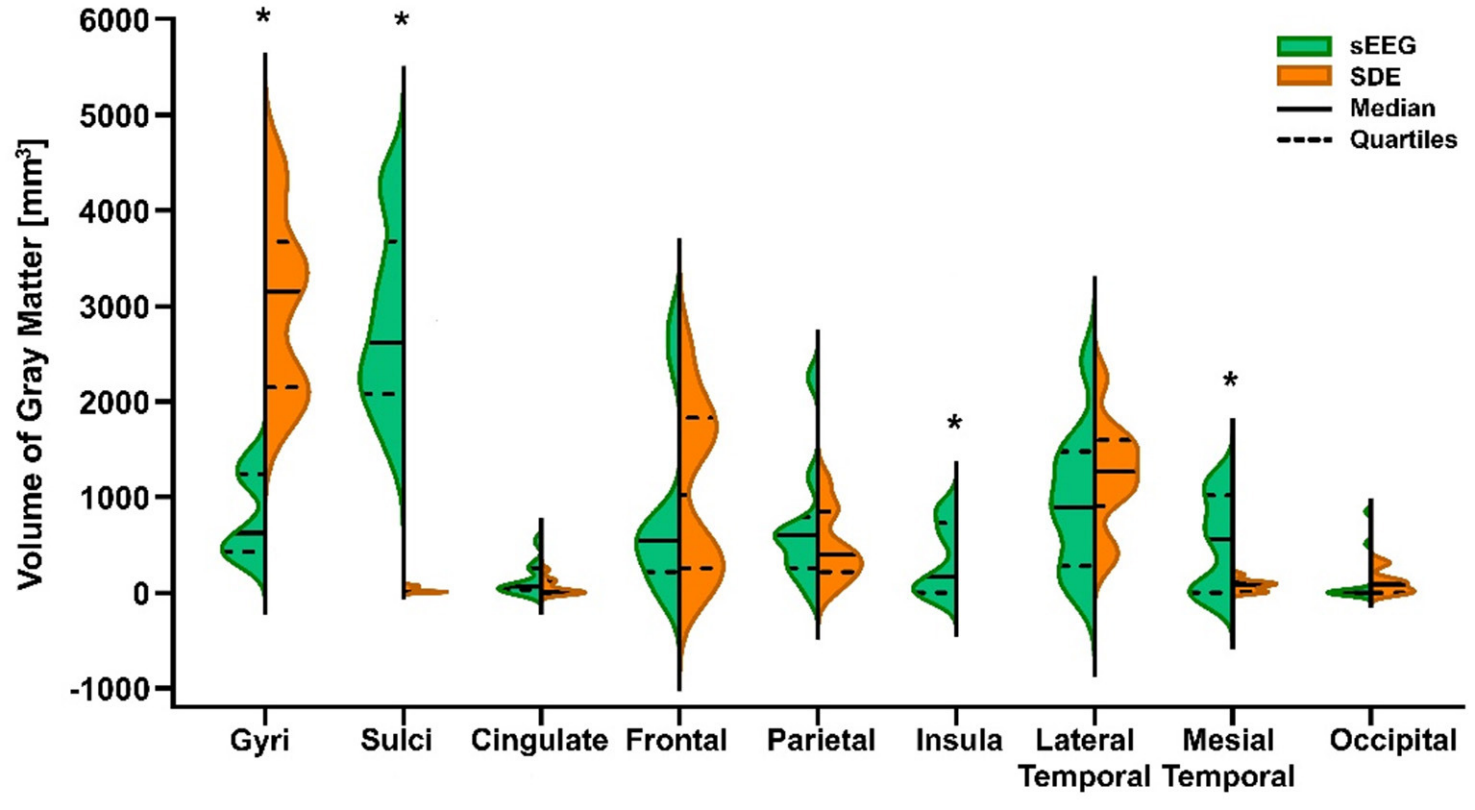
Violin plot of volume of gray matter recorded by SEEG and SDE implants in gyri, sulci, and 7 cortical regions of interest (ROIs) for the entire cohort. Statistically significant differences (*) between pair-matched SEEG and SDE cases were found in gyri (paired *t*-test, *p* < 0.0001), sulci (paired *t*-test, *p* < 0.0001), insula cortex (Wilcoxon test, *p* = 0.0156), and mesial temporal cortex (Wilcoxon test, *p* = 0.0273). Increased sampling over the lateral temporal lobe with SDE was not statistically significant.

A paired *t*-test showed significant differences in the recording volume of gray matter in gyri (*t* = 7.652, *p* < 0.0001) and sulci (*t* = 9.401, *p* < 0.0001) between paired SEEG and SDE cases. Comparison of the volume of gray matter recorded in ROIs between the paired cases showed statistically significant increases in sampling of the insula (Wilcoxon test, *p* = 0.0156) and mesial temporal cortex (Wilcoxon test, *p* = 0.0273) in the SEEG group. Wilcoxon tests in other ROIs were not significant (Figure 2).

### Spatial Pattern Distribution Analysis

**Figure 3:**
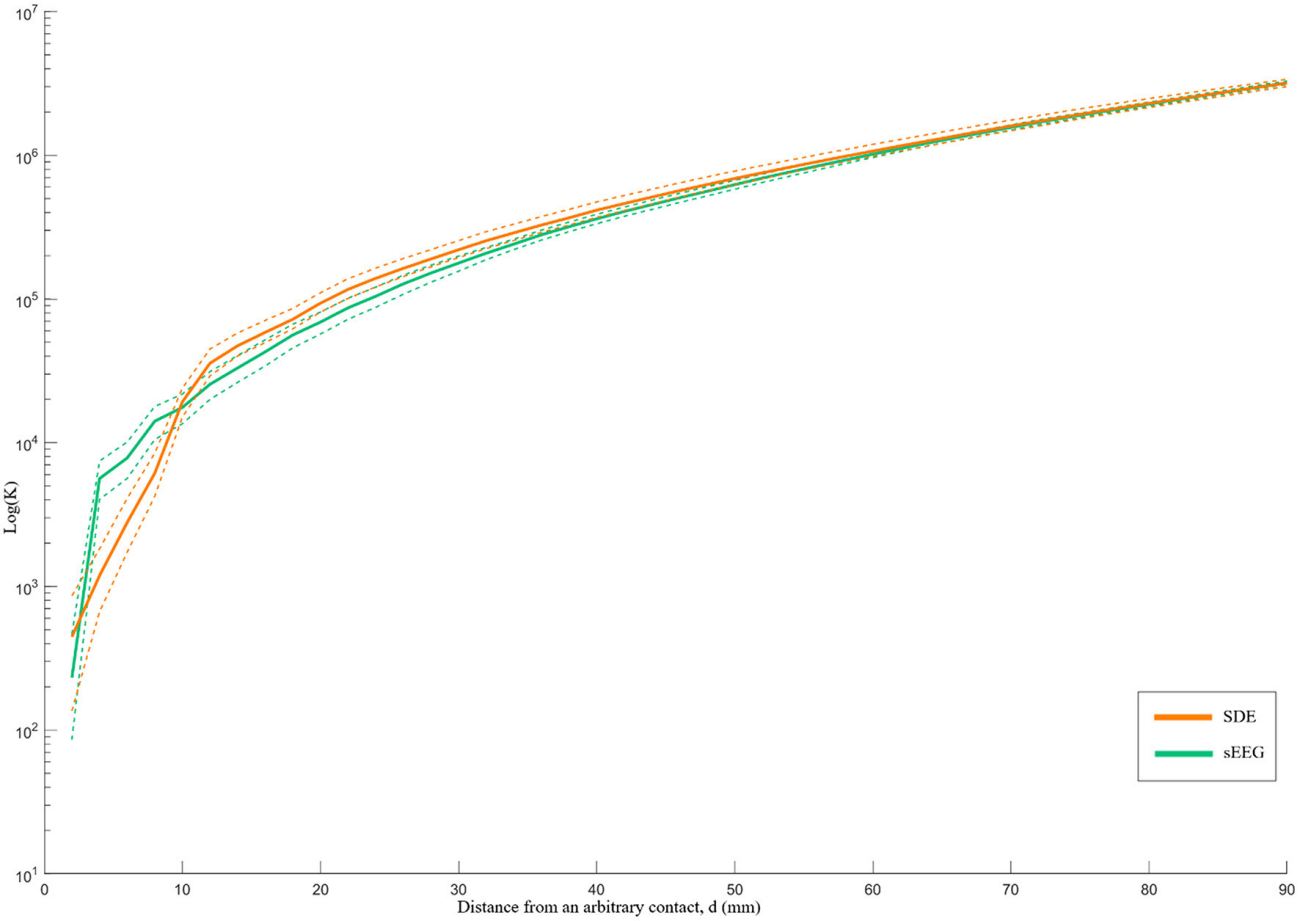
Ripley's k-function for SEEG and SDE over distance *d*. The Y-axis represents the K-function values on a logarithmic scale, while the X-axis represents the distance from an arbitrary contact in millimeters. The dashed lines represent the 95% confidence intervals of the k-function values. SEEG implants had denser coverage than SDE over distances <10 mm and sparser coverage than SDE over distances >10 mm.

Although both implant types demonstrate an overall pattern of clustering, the K-function analysis illustrates different patterns of spatial distribution. Specifically, relative to SDE implants, SEEG implants have greater clustering and denser local coverage (*d* < 10 mm) but greater dispersion with sparser coverage across larger distances (Figure 3). While the difference between K-function curves is statistically significant (*p*=0.039) for sampling within volumes <33cc (*d* < 20 mm), there was no difference identified over a larger range of volumes.

## Discussion

Historically, SDE implants or a combination of depth and SDE implants have been the most common methods for invasive long-term monitoring in patients with drug-resistant epilepsy; however, SEEG has gained popularity in North America over the past decade. Each modality has its own advantages and disadvantages, which should be considered during the selection of implant type.

### Comparison of SEEG and SDE Implantations

One question that has yet to be answered is if both methods provide similar spatial sampling. Our preliminary analysis demonstrates that while the mean sampling volume per contact was not different between SEEG and SDE implants, the total number of implanted contacts per paired cases was greater in SEEG subjects, leading to a greater overall volume of gray matter recording. Based on the average recording volume per gray matter contact and the percentage of gray matter contacts per implant, we estimate that 1.4 times as many SEEG contacts are needed to cover the same total recording volume as a single SDE contact. As such, this comparison is highly dependent on the number of electrodes implanted and institutional implantation strategy preferences. At the same time, the need for more electrodes implanted with SEEG may be justified in the setting of its known safety profile and its minimally invasive nature (10). While the number of implanted SEEG electrodes may be an institutional preference, we have attempted to control for the effects of different implantation strategies with our pairwise comparison between SEEG and SDE implants for matching pre-implantation hypotheses.

In addition to differences in the volumes of gray matter recording, we identified differences in the spatial distribution of recording between the two implant types. While SEEG implants predominantly sampled sulcal gray matter, SDE implants predominantly sampled the gray matter over the cortical convexity. The limited sulcal sampling ability of SDE denotes a clear disadvantage given that approximately two-thirds of brain gray matter lies within the sulci (15). In addition, lesions such as focal cortical dysplasias (FCDs) tend to reside at the depths of sulci (36) and may be missed with SDE recordings. Given that some of MRI-negative epilepsies may be explained by FCDs (37), the ability to record from sulcal depths may be of particular importance in MRI-negative explorations. SEEG not only excels at recording from the bottom of sulci, but also provides safe means of sampling deeper brain areas such as the insula, the cingulate gyrus, and the mesial temporal structures, which are commonly parts of the epileptogenic networks of interest (38). Suspected functional network involvement has been previously described as an indication for SEEG over SDE (39). Of note, in the present study, coverage of the insular gray matter was only possible in the SEEG cohort. Although sampling of deep structures is possible with SDE, it requires addition of depth electrodes or extensive dissection of fissures (40).

Lastly, we quantified different patterns of spatial coverage between SDE and SEEG implants. The spatial pattern analysis demonstrated that SEEG provides tight clusters of gray matter sampling – particularly for volumes < ~4cc (*d* < 10 mm). For this analysis, standard electrodes with 10 mm spacing between centers of contacts were used for the SDE subgroup; and electrodes with 3.5 mm spacing between centers of contacts were used in the SEEG subgroup. It is therefore logical that SEEG provides higher spatial resolution recordings within a distance of 10 mm, but yields more disperse coverage at distances beyond 10 mm. It should be noted that this comparison is specific to standard intracranial electrode designs and will be directly affected by using electrodes with different contact spacing. For example, wider spacing between SEEG contacts will lead to a coverage pattern closer to that of SDE. Ultimately, the Ripley's K-function analysis quantifies the visual differences in patterns of gray matter coverage that can also be seen in Figure 1. Qualitatively, one can recognize that SEEG implants generally consist of discrete areas of dense local coverage scattered across the brain; while SDE implants cover more cortical surface areas with lower density recording. For this reason, SEEG may be better suited for interrogation of specific nodes of diffuse networks, while SDE may be better suited for investigation of large superficial cortical regions.

The differences in volumes and patterns of gray matter coverage help to highlight the strengths and weaknesses of each implant type. In general, SDE has been preferred for language mapping because the contacts systematically cover larger cortical surface area, thereby allowing for more comprehensive stimulation testing and straightforward delineation of eloquent areas (41). Meanwhile, it has been felt that SEEG is suboptimal for language mapping because of the spatial separation between electrodes and overall coverage of the cortical surface. This limitation can be addressed by implantation of several SEEG electrodes in a tightly spaced three-dimensional grid over the area of interest (42). Such an approach provides comparable spatial resolution over the cortical surface, but it requires a number of electrodes to be implanted through presumed eloquent cortex. Additionally, since SEEG is associated with greater white matter sampling, accurate functional mapping of subcortical structures associated with these gray matter regions is possible with this modality (43). Overall, published evidence supports safe and feasible language mapping with electrical stimulation using SEEG (44).

Given its significant advantage of sulcal recordings, SEEG should be used when there is sufficient concern for an EZ at the depth of a sulcus – such as with focal cortical dysplasias. SEEG may also be more appropriate in patients who require more widespread spatial sampling and three-dimensional mapping of epileptic networks that may extend beyond the hypothesized EZ. In such scenarios, a clear hypothesis is necessary to derive a meaningful implantation strategy that is capable of interrogating different nodes of a presumed epileptic network. It has been suggested that the inability of SDE implants to interrogate networks in this manner can sometimes lead to false localization of epileptogenic activity – as the limited area of contiguous recording might represent a propagation zone and fail to recognize a clinically-silent onset zone (45).

### Limitations

One limitation of this study is a lack of consensus about the volume of spatial sampling around an implanted contact. Previous studies have reported a large range of experimental and theoretical estimates of the spatial reach of LFPs, which is due to the fact that LFP recording depends on multiple sources, different brain regions, and different brain states (29, 46, 47). While it is widely accepted that the actual recordings are limited to a very small local area around the contact, the definition of the spatial extent of the LFP is disputable. It is unclear whether the LFP represents the activities of a few neurons adjacent to the contact or a larger connected network (47). Given that the purpose of this study is to compare the *relative* recording volume between two types of iEEG implants, we applied a recent LFP model suggested by Maling et al., with an assumption of the same spatial volume recorded by the contacts of SEEG and SDE (31). This approach certainly serves as an oversimplification but is also reasonable given our current understanding of the capabilities of electrode recording.

Another limitation is the accuracy of the contact localizations, particularly for SDE implants, which are subject to significant brain shift and cortical deformations. Given the large numbers of contacts (72–298 contacts) per patient and the relatively large recording volume model (5 mm diameter) used in this study, errors from the inaccurate localization are unlikely to significantly affect our results.

Although we sought to match SEEG and SDE subjects based on clinical presentations and implant strategies, the retrospective nature of this study makes it inherently susceptible to selection bias. Certain clinical features or radiographic findings may have led to a preference of one type of implant over the other. Furthermore, as a single-center study, institutional preferences regarding the number and specific locations of electrodes implanted directly impact the analyses performed in the current study. While the recording volume per gray matter contact and the percentage of sulcal coverage are unlikely to be affected, total volumes of gray matter recording and the patterns spatial distribution can certainly be impacted by the number of implanted SEEG electrodes. As such, the generalizability of the latter findings may be more limited, as implantation strategies and preferences vary among epilepsy centers, which may be due to different experiences, skills, and techniques.

## Conclusion

In this study, we compared estimated volumes of gray matter recorded by SEEG and SDE. Average recording volumes per electrode contact were similar for SEEG and SDE, but SEEG allowed for greater overall volumes of sampling as more electrodes were routinely implanted. The primary difference between the two modalities lies in the location of gray matter than can be sampled. These results provide a better insight into the capabilities of SEEG and SDE and may help epilepsy centers make an informed decision about which intracranial monitoring technique is more appropriate for each epilepsy patient.

## Data Availability Statement

The raw data supporting the conclusions of this article will be made available by the authors, without undue reservation.

## Ethics Statement

The studies involving human participants were reviewed and approved by Thomas Jefferson University IRB. Written informed consent for participation was not required for this study in accordance with the national legislation and the institutional requirements.

## Author Contributions

CW contributed to the conception and design of the study. CM, JM, and MT collected and organized the database. MT and JM performed the data processing and statistical analysis. MT wrote the first draft of the manuscript. JM and CW wrote sections of the manuscript. All authors contributed to the article and approved the submitted version.

## Conflict of Interest

The authors declare that the research was conducted in the absence of any commercial or financial relationships that could be construed as a potential conflict of interest.
