# Supplementary material for: Gray Matter Sampling Differences Between Subdural Electrodes and Stereoelectroencephalography Electrodes
Source: Front Neurol. 2021 Apr 27;12:669406. doi: 10.3389/fneur.2021.669406 (PMC8110924; doi:10.3389/fneur.2021.669406)
Supplement: Supplementary file 1 [file Image_1.pdf]

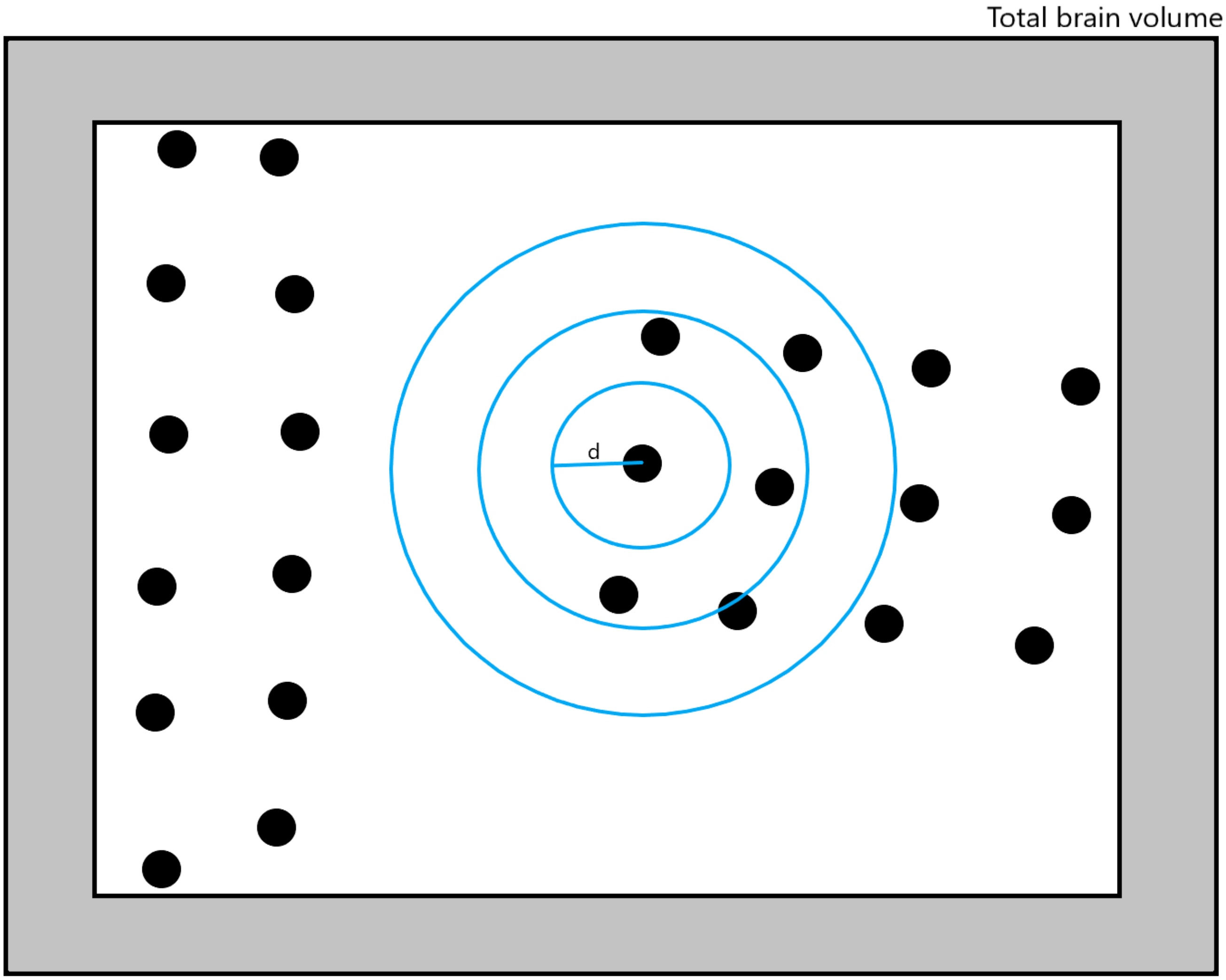

Supplementary Figure 1. A schematic representing Ripley's K-function calculation. The black solid circles represent implanted contacts. The numerator of the K-function is calculated by averaging the number of neighboring contacts within a radius *d* (in millimeters) [blue concentric circles] across all contacts of the implant. The denominator of total contact density serves a means of normalization and is calculated by dividing the total number of contacts implanted by the minimal space containing all contacts [smaller white rectangle], rather than the total brain volume [outer gray rectangle]. In this manner, the K-function can be calculated as a function of distance to characterize the clustering and spatial coverage of an implant.
